# Punishment in the public goods game is evaluated negatively irrespective of non-cooperators’ motivation

**DOI:** 10.3389/fpsyg.2023.1198797

**Published:** 2023-06-29

**Authors:** Yang Li, Nobuhiro Mifune

**Affiliations:** ^1^School of Informatics, Nagoya University, Nagoya, Japan; ^2^School of Economics and Management, Kochi University of Technology, Kochi, Japan

**Keywords:** punishment, public goods, third-party punishment, fear, greed, reputation

## Abstract

The evolution of human cooperation toward strangers remains puzzling. While the punishment of non-cooperators is a possible explanation, whether punishments can help cooperation evolve depends on how people evaluate punishers. Thus, it is of vital importance to elucidate the perception of punishers. Previous studies have found that punishment is evaluated differently in different contexts; punishers are negatively and positively evaluated in the public goods game with punishment (PGG-P) and in the third-party punishment game (TPP), respectively. To disentangle the mixed evidence, our research focused on motivations for non-cooperation and examined whether different motivations for non-cooperation could explain the inconsistent findings. We hypothesized that people positively evaluate punishers when they punish those who non-cooperated to exploit others (greed), e.g., in a TPP situation. Contrastingly, people negatively evaluate punishers when they punish those who non-cooperated to avoid exploitation (fear), e.g., in a PGG-P situation. If so, in either game, punishers would be evaluated positively in situations where greed dominates, and negatively in situations where fear dominates the non-cooperation motivation. To test this, we conducted two online experiments with hypothetical scenarios in which the decision-making order was used to manipulate the motivations of non-cooperators. The results of Study 1 (*N* = 240) using only PGG-P and Study 2 (*N* = 602) using both PGG-P and TPP showed that the non-cooperation motivation did not have a significant effect on the evaluations of punishers and non-punishers. Punishers (*vs* non-punishers) were evaluated negatively in PGG-P and positively in TPP regardless of the decision-making order of non-cooperation. We discussed the role of higher-order information in the evaluation of punishment.

## 1. Introduction

To form and maintain large-scale societies, it is essential for humans to cooperate with strangers, even in the absence of genetic or reciprocal relationships (Fehr and Fischbacher, 2003). However, the evolutionary mechanism of cooperation remains to be elucidated (Fehr and Rockenbach, 2004; Pennisi, 2005; Nowak, 2006; Apicella and Silk, 2019; Kay et al., 2020). Numerous scholars have focused on the role of punishment (e.g., Gintis, 2000; Fehr et al., 2002; Fehr and Gächter, 2002). Selfish individuals do not cooperate with strangers, but the punishment that reduces their benefits of non-cooperation will likely incentivize them to cooperate. Thus, cooperation with strangers can evolve in the presence of punishment. Yet, given that punishment is costly, individuals face a second-order social dilemma: self-regarding individuals avoid punishing others. Therefore, there is a need for explanations of how individuals solve the dilemma and, correspondingly, successful punishment facilitates the evolution of cooperation (Henrich and Boyd, 2001; Henrich, 2004).

Reputation provides one possible solution to the second-order dilemma problem (Brandt et al., 2003; dos Santos et al., 2011; Hilbe and Traulsen, 2012). Many mathematical simulations and experimental studies have shown that individuals who acquire a good reputation receive altruistic behavior and/or positive treatments from others (e.g., Nowak and Sigmund, 1998; Leimar and Hammerstein, 2001; Milinski et al., 2002; Panchanathan and Boyd, 2004; Ohtsuki and Iwasa, 2006; Barclay and Willer, 2007; Van Vugt and Hardy, 2010). In other words, punishment can evolve if punishers gain a good reputation and the corresponding benefits outweigh the costs of punishment. Such benefits may take various forms, including being well-regarded by others, being chosen as an interaction partner, and receiving resources because of punishment (Nelissen, 2008; Raihani and Bshary, 2015a; Jordan et al., 2016; Batistoni et al., 2022).

To investigate how people evaluate punishers, most studies have utilized two types of experimental economic games: the public goods game with punishment (PGG-P) and the third-party punishment game (TPP). Both games consist of a first stage that measures cooperative and noncooperative behavior and a punishment stage that measures punishing behavior toward opponents who have behaved noncooperatively in the first stage. In the PGG-P, a group of (typically four) individuals play a game in which each must decide whether and/or how much of their monetary endowment they will contribute to their group (i.e., toward the public good) in the first stage. The aggregated contribution is increased by the experimenter (e.g., doubled) and evenly distributed among the group members. Thus, individuals who do not cooperate (i.e., do not contribute to the public good) will earn a higher amount of money than those who do, as they retain more (or all) of their original endowments while also receiving an equal share of the aggregated public goods. If all members cooperate, this maximizes the collective earning. After the first stage, one of the four players is assigned the role of punisher, and must decide the extent to which they will reduce the earnings of non-cooperators by paying a cost from their earning, which otherwise will become the earnings of the punisher in the punishment stage. In the TPP, the first stage typically consists of a dictator game or prisoner’s dilemma. In the dictator game, one of the paired participants is the allocator and the other is the receiver, and the allocator decides how much of the endowment to allocate to the receiver. The receiver cannot reject the offer but, if any, accept the allocated money. In the prisoner’s dilemma game, the public goods game described above is played between two participants. Unlike the PGG-P, a third party who does not play the first stage decides whether and how much to punish the player in the game at their cost in the punishment stage. Usually, participants observing these situations rate the others who did or did not punish them in the punishment stage on items such as whether they think they are good or trustworthy.

There is an ongoing debate on whether punishment is evaluated positively or negatively (Jordan and Rand, 2020; Batistoni et al., 2022; For a review, see Raihani and Bshary, 2015b; Redhead et al., 2021). Our assessment of existing studies suggests that punishment is most often evaluated negatively in the PGG-P, but is usually evaluated positively in the TPP. For example, Kiyonari and Barclay (2008) found that PGG-P players who punished non-cooperators were evaluated more negatively than those who did not and were not rewarded. Moreover, Ozono and Watabe (2012) reported that punishers were not selected as partners to play a subsequent experimental economic game (but also see Barclay, 2006; Horita, 2010). By contrast, Nelissen (2008) found that TPP punishers were more positively evaluated than non-punishers and more likely to be selected as partners in subsequent games; in line with this, a number of studies have shown that TPP punishers are more likely to be rewarded and trusted than non-punishers (Raihani and Bshary, 2015a; Jordan et al., 2016; Batistoni et al., 2022).

There are some possible explanations for the discrepancy in the evaluation of punishers in PGG-P and TPP, e.g., the punisher’s involvement in cooperation games or the motives for non-cooperation in the first stage; the punishers in PGG-P have been harmed by a non-cooperator in a public goods game, and their punishment may be perceived to be vengeful. Contrastingly, a non-cooperator has not harmed the punisher in TPP and therefore the punishment is not considered vengeful but altruistic. Thus, PGG-P punishment, which is revengeful punishment, is evaluated negatively, while TPP punishment, which is altruistic punishment, is evaluated positively. Mifune et al. (2020), however, did not find experimental evidence for the explanation. They used the PGG-P and manipulated whether the potential punisher played the public goods game in the first stage, predicting the punisher would be positively evaluated as in TPP if the punisher did not participate in the first stage. Yet, they found no evidence to support their hypothesis. This result suggests that the different evaluation between the PGG-P and TPP cannot be attributed to the punisher’s involvement in the first stage game.

The current study tests the second possibility described above, i.e., the possibility that the discrepancy in the evaluation of punishers between the PGG-P and TPP may be attributable to the inference of motives for non-cooperative behavior. In the context of a social dilemma, motives for non-cooperation are broadly categorized as either fear or greed (Dawes, 1980; Simpson, 2003). In this context, fear-based non-cooperation emerges under the expectation that others will not cooperate, while greed-based non-cooperation emerges under the expectation that others will cooperate; in other words, the goal of fear-based non-cooperation is the defense against the expected exploitation, but that of greed-based non-cooperation is the exploitation of others. Importantly, previous studies found that fear-based non-cooperation is less likely to receive negative evaluations than greed-based non-cooperation (Horita and Yamagishi, 2010). Non-cooperative behavior in a typical TPP that employs a dictator game can only be motivated by greed, as the role of receiver does not have a chance to cooperate or defect, thus eliminating the influence of fear of exploitation on the decision-making of allocators (Yamagishi and Mifune, 2008). In contrast, while non-cooperation in public goods games could include both fear and greed, fear should be a more dominant motivation for non-cooperation (Yamagishi, 1986; Yamagishi and Sato, 1986; De Cremer, 1999). If exploitative non-cooperation (greed) is evaluated negatively and non-cooperation in defense of exploitation (fear) is evaluated positively, then the punishment for the former (i.e., punishment in the TPP) would be evaluated positively, and punishment for the latter (i.e., punishment in the PGG-P) negatively.

Numerous studies on the evolution of cooperation through indirect reciprocity have revealed that people evaluate other’s behavior not only based on first-order information, i.e., whether others cooperate or defects (Wedekind and Milinski, 2000), but also on second-order information, i.e., whether they cooperate or defect toward one with a good reputation or a bad reputation (Swakman et al., 2016; Okada et al., 2018; Yamamoto et al., 2020). These studies suggest that individuals use higher-order information for the evaluation of cooperation. People may utilize such higher-order information to evaluate punishment as well. That is, people may take into account whom others punish, those who displayed fear-based or greed-based non-cooperation.

In the current research, therefore, we predicted that the evaluation of punishers would be influenced by the inferred motive for non-cooperation of the punished. In Study 1, using the PGG-P only, we manipulated the non-cooperator’s motive through the order of decision-making, such that a non-cooperative decision was made under one of the following conditions: (1) all players make decisions at the same time (simultaneous condition; SIM), (2) non-cooperation precedes other decisions in a sequential game (FIRST condition), and (3) non-cooperation followed cooperative decisions in a sequential game (LAST condition). For all conditions, it is important to note that there was only one non-cooperator and the other players all cooperated. In the FIRST condition, the first player (non-cooperative target) chooses non-cooperation without knowing whether others would cooperate. Thus, non-cooperation can be motivated by both fear and greed, but fear should at least hold a stronger influence in the FIRST condition compared to the LAST condition (Horita and Yamagishi, 2010). In the LAST condition, the other players already cooperated, and the non-cooperator should be motivated by greed, not fear. From a logical standpoint, in the SIM condition, non-cooperation should also be subject to a stronger influence of fear than that induced in the LAST condition. Yet, it is not clear whether such an effect would be stronger than that induced in the FIRST condition. Thus, we hypothesize as follow.

*Hypothesis 1-1:* In the PGG-P, non-punishers will be evaluated more positively than punishers in the FIRST condition; by contrast, punishers will be evaluated more positively than non-punishers in the LAST condition.

## 2. Study 1

### 2.1. Materials and methods

Study 1 was reviewed and approved by the ethical committee at Kochi University of Technology. In accordance with the Declaration of Helsinki, all participants provided written informed consent.

#### 2.1.1. Design

In Study 1, we had a 3 (decision-making order of non-cooperator: SIM vs. FIRST vs. LAST) × 2 (evaluation target: punisher vs. non-punisher) mixed design, with the former being a between-subject factor. The order of the presentation of the two within-subject conditions was randomized and counterbalanced.

#### 2.1.2. Participants

We initially planned to recruit 100 participants for each condition (300 total) from a Pool of Japanese university students, all of whom would receive a fixed fee of 500 yen. However, we could not recruit as many participants as we expected as the experiment was conducted during their final examination period. Thus, we had to end the recruitment process earlier and we had a final sample of 240 participants (128 females, 108 males, four neither; mean age = 20.6; SD = 1.4). The proportion of gender and mean of age were not different among conditions (gender: *χ^2^* (2) = 3.9765, *p* = 0.41; age: *F* (2, 237) = 0.33, *p* = 0.72).

#### 2.1.3. Procedure

We conducted a vignette experiment using Qualtrics. First of all, participants were told that this experiment was fictive. More specifically, they were told that they would not play any games with others but would just read a scenario and answer some questions. The fictive scenario given to participants read as follows; “Some individuals participated in a past experiment. The experiment consisted of two stages. In the first stage, four players each received 1,000 yen as an endowment, and decided how much of the endowment they would give to the group. Any amounts of money that they did not give were their earning, while the total amount contributed to the group was doubled by the experimenter and then equally distributed among all players. It was a one-shot decision-making.” And participants read that only one player kept their initial amount (i.e., non-cooperator), while the others contributed all of their endowment to the group.

We manipulated the order of the exchanges in the first stage. In the SIM condition, all four players simultaneously decided how much money they would contribute to the group. In the FIRST and LAST conditions, the decisions were made sequentially. In the FIRST condition, the first one player kept their full endowment for themselves, then the other three players contributed all of their endowment to the group. In the LAST condition, the first three invested all of their endowment to public goods, and then the last player kept all for oneself.

In the second stage, all four players were each given the additional endowment of 500 yen, and they were then asked to decide whether to use the money to reduce the payoff of other players, knowing that any money that was not used for punishment would be their earning. Three times the amount invested in punishment was deduced from the punished. Deduced money was collected by the experimenter. It was then explained that one of the three cooperators in the first stage (the punisher) used 500 yen to reduce the money of the non-cooperator (also in the first stage), while the others (non-punishers) did not use any money.

The participants read one of the three scenarios (the SIM, FIRST, or LAST condition), and answered questions measuring their impressions and evaluations of the punisher and the non-punishers (all of whom were cooperators in the first stage) as well as their intention to cooperate with them. The evaluation was measured using three factors, including trustworthiness, likability, and kindness. For the intention to cooperate, participants were asked two questions: “If the person asks you for directions, would you be willing to help?” and “If you see the person struggling to climb upstairs with large luggage, would you be willing to help?” All responses were rated on a 7-point scale ranging from “1: Not at all” to “7: Very much.” The alpha coefficients were 0.85 for impressions of the punisher and 0.79 for impressions of the non-punisher. The alpha coefficients for intention to cooperate were 0.85 and 0.81 for the punisher and non-punishers, respectively. The alpha coefficient is a measure of internal consistency, and the values shown in the study all indicate a high degree of internal consistency. The mean values of each set of questions were computed and used as indices in the following analysis.

After giving their impression evaluations and indicating their intention to cooperate, participants were asked about their inference of thoughts of non-cooperators. Details on the related items are available in the Supplementary materials.

### 2.2. Results

**Figure 1:**
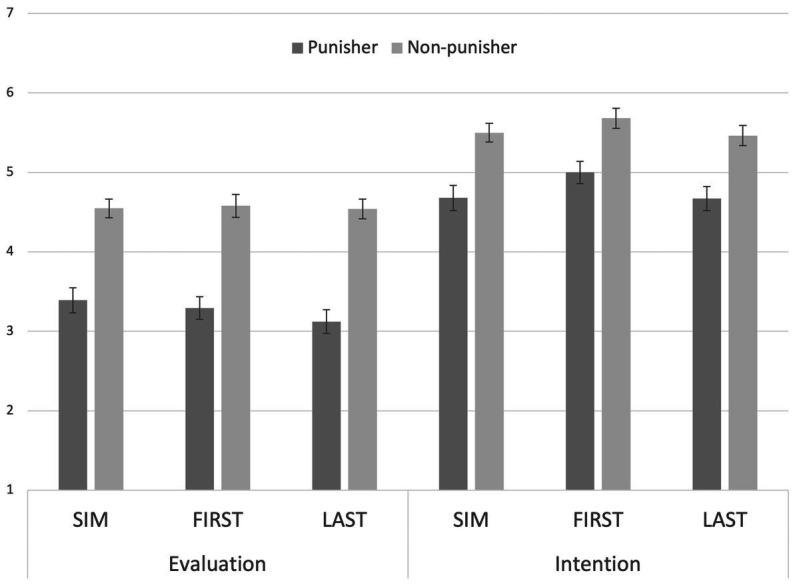
Mean impression evaluations and cooperative intentions in Study 1. SIM means simultaneous decision, FIRST means the first decision, and LAST means the last decision of non-cooperation in the first stage. Error bars indicate standard errors.

Figure 1 shows the mean values of impression evaluation and the intention to cooperate in each condition. We conducted a mixed-factor 3 (order) × 2 (target) MANOVA with impression evaluation and intention to cooperate as dependent variables. The analysis revealed a non-significant main effect of the order (Wilks’s Lambda = 0.9772, *p* = 0.71, partial η2 = 0.022) and a non-significant interaction effect (Wilks’s Lambda = 0.9985, *p* = 0.836, partial η2 = 0.002). Yet, the main effect of target was significant (Wilks’s Lambda = 0.6265, *p* < 0.001, partial η2 = 0.37), suggesting that non-punishers were rated more positively than punishers. These results did not support our hypothesis.

### 2.3. Discussion

The result of Study 1 showed that punisher evaluations were not influenced by non-cooperators’ motivation. In the PGG-P, players who punished the non-cooperator were evaluated more negatively and also not likely to receive help than those who did not punish, regardless of the order of the non-cooperation decision. There were, however, three limitations in Study 1. First, it remains unclear whether the finding would be specific to the PGG-P or can be generalized to other games such as the TPP. Second, we did not reach our planned sample size. Third, the negative evaluation could be explained by the possibility that the punisher was perceived to deviate from non-punishing norm; there were three cooperators and one of them exerted punishment, which leads participants to assume the punisher violated the descriptive norm.

## 3. Study 2

The aim of Study 2 was to investigate whether non-cooperation motivation affected the evaluation of punishers in the PGG-P and TPP. To achieve a direct comparison of the two games, we used the TPP with the prisoner’s dilemma game because the dictator game does not involve fear as a potential motivation for non-cooperation. In addition, whether the punisher was a second-or third-party was confounded when comparing PGG-P and TPP because punishers participated in the first stage of PGG-P in Study 1. Therefore, we had a third-party punisher in the PGG-P, i.e., the punisher did not play the public goods game. Mifune et al. (2020) showed that there was no difference in the evaluation in the PGG-P when the punisher plays as a third-party or second-party. In addition, we controlled for the influence of descriptive norms on the evaluation of punishment by setting up a situation in which there was one punisher and one non-punisher in both games. We thus had the following two hypotheses.

*Hypothesis 2-1:* In the PGG-P, participants positively evaluate and show intention to cooperate with non-punisher than punisher, regardless of the order condition.

*Hypothesis 2-2:* In the FIRST and LAST conditions of the PGG-P, participants positively evaluate and show intention to cooperate with non-punisher than punisher. In the FIRST condition of the TPP, participants more positively evaluate and show increased intention to cooperate with non-punisher than punisher, while in the LAST condition of the TPP, participants positively evaluate and show intention to cooperate with punisher than non-punisher.

We predicted similar results as in Study 1 with respect to PGG-P and set hypothesis 2-1. We also predicted that the manipulation of the non-cooperation motivation would be effective in TPP and set Hypothesis 2-2.

### 3.1. Materials and methods

Study 2 was reviewed and approved by the ethical committee at Kochi University of Technology. In accordance with the Declaration of Helsinki, all participants provided informed consent.

We preregistered Study 2 in the open science framework (https://osf.io/ju6vn). We would like to note that the first version of preregistration contained some errors in the SAS code for the analysis, which could not be analyzed properly, and we corrected our codes. This was the only deviation from the preregistration.

#### 3.1.1. Design

In Study 2, we had a 2 (Game: PGG-P vs. TPP) × 3 (Order of non-cooperative decision: SIM vs. FIRST vs. LAST) × 2 (Target: punisher vs. non-punisher) mixed design. Target was a within-subject factor with the order of the presentations being counter-balanced, while game and order of non-cooperative decision were between-subject factors.

#### 3.1.2. Participants

Since there was no significant interaction effect in Study 1, we predicted that the effect size of the interaction in Study 2 (Hypothesis 2-2) may have also been small. Specifically, we conducted a *a priori* power analysis with PANGEA (expected effect size: Cohen’s d = 0.2, statistical power = 0.95, alpha = 0.05) and found that the total required sample size was 600. As such, we recruited a total of 602 participants from the Japanese crowdsourcing service Lancers.^1^ Due to an unexpected error in the configuration, we did not obtain data on gender or age. However, since participants are randomly assigned to conditions by the Qualtrics system, it is unlikely that participant characteristics differ between conditions.

#### 3.1.3. Procedure

Similar to Study 1, Study 2 was an online survey using Qualtrics. The participants read a fictitious scenario about a hypothetical experiment, either involving a PGG-P with punishment (the PGG-P condition) or TPP with prisoner’s dilemma and punishment (the TPP condition). After reading the scenario, they answered questions measuring their impression evaluations and intentions to cooperate with the punishers and non-punishers.

In the PGG-P condition, the game consisted of five players. Specifically, four players (A, B, C, and D) made decisions in the first stage (PG stage), while the remaining players (E or F) engaged in the second stage (punishment stage). The instruction of the public goods stage was identical to that in Study 1, as was the manipulation of the decision-making order. The punishment stage was also similar to Study 1, except that the punisher/non-punisher (E/F) did not participate in the first stage (i.e., not a PGG-Player). Participants were presented with two scenarios in a randomized order: one scenario in which the observer (E) punished the non-cooperator, and the other in which the observer (F) did not. Participants then responded to questions measuring their impression evaluations and intentions to cooperate with the punisher or non-punisher.

In the TPP condition, a prisoner’s dilemma with an observer was used in the first stage. Players A and B received a fixed show-up fee of 500 yen, while an additional 500 yen was given as an endowment in the first stage. Each yen that they gave to the counterpart was doubled by the experimenter, before it was received by the counterpart. This exchange occurred only once. Similar to the PGG-P condition, the order of decision-making in the first stage of the TPP condition was manipulated. In the SIM condition, the two players made decisions simultaneously, wherein one player offered the full amount and the other kept the full amount. In the FIRST condition, the first player kept their entire sum of money, while the second player offered their entire sum of money to the first player. In the LAST condition, the first player offered their entire sum of money to the second player, and the second player, who knew that their counterpart had cooperated, kept the endowment for themselves.

In the TPP punishment stage, the observer in the first stage (i.e., player C or D) made the decision; this player was also given a fixed show-up fee of 500 yen, and an additional endowment of 500 yen. They then decided whether to use the endowment to deduct money from the players in the first phase (A or B). As in Study 1, three times the amount invested in punishment was deduced from the punished, and the remaining amount of money after punishment would be added to Player C/D’s earnings. As in the PGG-P condition, participants were presented with a scenario in which player C decided to punish (i.e., use all 500 yen to deduct money from the non-cooperator), and another scenario in which the observer (this time, player D) decided not to punish. The order of these scenarios (i.e., punisher or non-punisher) was randomized. Finally, participants responded to questions measuring their impression evaluations and intentions to cooperate with the punisher or non-punisher, respectively.

#### 3.1.4. Measurements

In Study 2, we used the items from Study 1 for the impression evaluations and intention to cooperate. The alpha coefficients were 0.92 for the impression evaluation of the punisher and 0.92 for the impression evaluation of the non-punisher. The alpha coefficients for intention to cooperate were 0.92 and 0.92 with the punisher and non-punishers, respectively. These values indicated high internal consistency across all measures. We also included the following items for exploratory purposes: (a) inference of thought of non-cooperators, (b) evaluations of the non-cooperator in the first stage (using the same items for the punisher and non-punisher), (c) intentions to punish the non-cooperator if participating in the hypothetical experiment, and (d) extent to which participants cared about equality among players in the first stage, equality among first-stage and second-stage players, and whether the non-cooperator was punished.

### 3.2. Results

#### 3.2.1. Hypothesis testing

**Figure 2:**
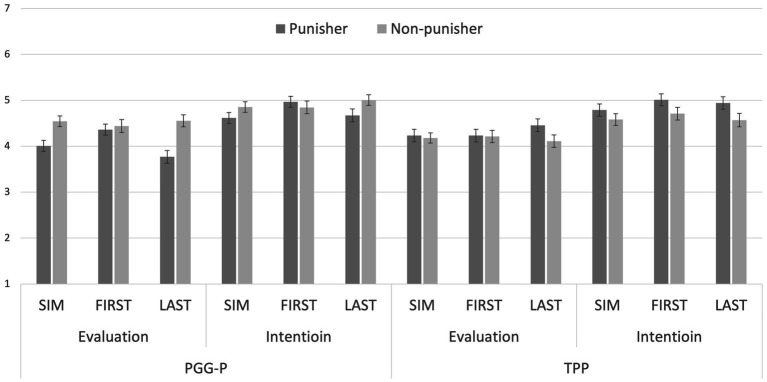
Mean impression evaluations and cooperative intentions in Study 2. SIM means simultaneous decision, FIRST means the first decision, and LAST means the last decision of non-cooperation in the first stage. Error bars indicate standard errors.

Figure 2 shows the mean values of the impression evaluation and the intention to cooperate in each condition. To test Hypothesis 2-1, we conducted a 3 (Order) × 2 (Target) mixed-factor MANOVA with impression evaluation and intention to cooperate as dependent variables, using data from participants in the PGG-P condition. We found a significant main effect of target (Wilks’s Lambda = 0.9702, *p* = 0.003, *partial η^2^* = 0.03), indicating that the non-punisher (*vs* punisher) was positively evaluated. There was no significant main effect of order (Wilks’s Lambda = 0.9555, *p* = 0.099, *partial η^2^* = 0.045) and there was no significant interaction effect between order and target (Wilks’s Lambda = 0.9813, *p* = 0.006, *partial η^2^* = 0.019). These results were consistent with those in Study 1, supporting Hypothesis 2-1.

To test Hypothesis 2-2, we conducted a mixed-factor 2 (Game) × 3 (Order) × 2 (Target) MANOVA with the impression evaluation and the intention to cooperate as dependent variables. The results showed a significant main effect of game (Wilks’s Lambda = 0.9771, *p* = 0.008, partial η^2^ = 0.023) and a significant game x target interaction effect (Wilks’s Lambda = 0.9789, *p* < 0.001, partial η^2^ = 0.021). However, the predicted three-way interaction was not significant (Wilks’s Lambda = 0.9921, *p* = 0.095, partial η^2^ = 0.008). Furthermore, there were no significant main effects of order (Wilks’s Lambda = 0.9901, *p* = 0.656, partial η^2^ = 0.01) and target (Wilks’s Lambda = 0.9993, *p* = 0.534, partial η^2^ = 0.001). Nor were there significant interaction effects between game and order (Wilks’s Lambda = 0.9789, *p* = 0.122, partial η^2^ = 0.002) or between order and target (Wilks’s Lambda = 0.9971, *p* = 0.418, partial η^2^ = 0.003). These results did not support Hypothesis 2-2.

To further probe the significant interaction effect between game and target, we conducted a one-way MANOVA with target as a single factor separately for participants in the PGG-P and those in the TPP. The results showed that punishers were less positively evaluated than non-punishers in the PGG-P (Wilks’s Lambda = 0.9708, *p* = 0.003, partial η^2^ = 0.029), but more positively evaluated than non-punishers in the TPP (Wilks’s Lambda = 0.9858, *p* = 0.038, partial η^2^ = 0.014).

#### 3.2.2. Exploratory analysis

**Figure 3:**
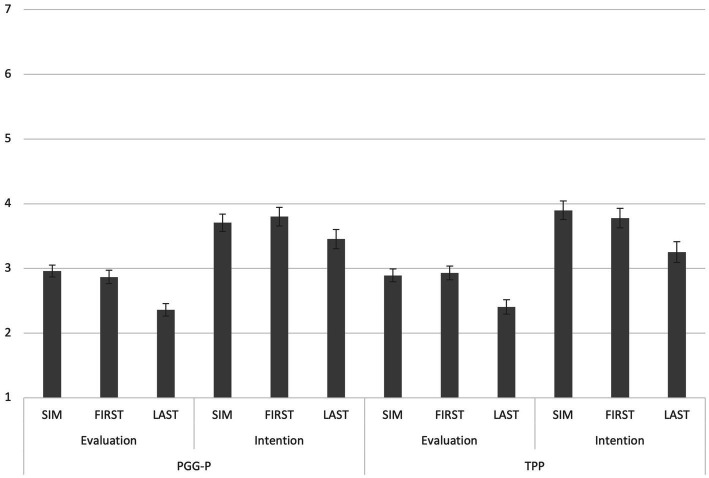
Mean impression ratings and cooperative intention toward a non-cooperator. SIM means simultaneous decision, FIRST means the first decision, and LAST means the last decision of non-cooperation in the first stage. Error bars indicate standard errors.

Figure 3 shows the mean values of the impression evaluation and the intention to cooperate in each condition. 2 (Game) × 3 (Order) MANOVA with evaluation ratings and cooperation intention toward the non-cooperator (note, not the punisher) set as dependent variables showed a significant main effect for order (Wilks’s Lambda = 0.9430, *p* < 0.001, partial η2 = 0.029), non-significant main effect for game (Wilks’s Lambda = 0.9999, *p* = 0.96, partial η2 = 0.000), and non-significant interaction effect (Wilks’s Lambda = 0.9919, *p* = 0.303, partial η2 = 0.004). To interpret the main effect of order, we standardized the evaluation ratings and cooperation intention, respectively, (Mean = 0, SD = 1), then used the mean of the two variables as an attitude score. A multiple comparison with Holm method revealed that the attitude score in the LAST condition was lower than those in both the FIRST condition (*p* < 0.001, *d* = 0.446) and SIM condition (*p* < 0.001, *d* = 0.467). There were no significant differences in scores between the FIRST and SIM conditions (*p* = 0.839, *d* = 0.02). Thus, participants felt that the non-cooperator in the LAST condition was behaving as a “bad” person.

## 4. General Discussion

The motive for non-cooperation did not affect how punishments were evaluated in both of the two studies. In Study 2, our exploratory analyses on attitude scores (i.e., impression evaluations and the intention to cooperate) showed that participants felt negatively toward non-cooperators in the LAST condition (*vs* the FIRST and SIM conditions) in both the PGG-P and TPP. This result suggests that greed-based non-cooperation was evaluated more negatively than fear-based non-cooperation. Nevertheless, participants negatively evaluated players who punished the non-cooperator in PGG-P, regardless of whether the non-cooperator had knowledge of the decisions made by other players. Similarly, the order of the non-cooperation decision did not influence how punishments were evaluated in the TPP. In other words, punishment in PGG-P was negatively evaluated even when non-cooperation was based on greed, and punishment in TPP was positively evaluated even when non-cooperation was based on fear. Our studies suggest that punishment evaluations were not affected by the estimation of the motive or the evaluation of non-cooperation.

Our studies offer relevant underpinnings to the empirical literature on how people use social information to decide whether to cooperate with others. Various previous studies examined whether indirect reciprocity assures the evolution of cooperation towards strangers when only first-order information is referred to, or otherwise second-or higher-order information needs to be utilized (e.g., Nowak and Sigmund, 1998; Leimar and Hammerstein, 2001; Ohtsuki and Iwasa, 2006). To put it concretely, imagine a scenario involving four individuals: A, B, C, and D, each of whom has to decide whether to give resources to the others. If A gives resources to B based on the information that B has already given his/her resources to C, A is referring to first-order information. If A gives resources to B based on the information that B has already given his/her resources to C, who had given resources to D, A is referring to second-order information. A number of previous studies showed that people refer to second-order information (Swakman et al., 2016; Okada et al., 2018; Yamamoto et al., 2020), but some other ones showed that people refer only to first-order information (Milinski et al., 2001). Using the example scenario, our study settings can be abstracted as follows: A (participants) evaluated B’s behavior (punish or not punish) toward C who did not cooperate towards D who had already cooperated (the LAST condition), or C who did not cooperate towards D who had not yet made any cooperation decisions (the SIM and FIRST conditions). Participants in the current study could thus use first-to third-order information to make their evaluation of punishers and non-punishers. The results indicate that the evaluation of punishment was not dependent on why the punished did not cooperate, while the evaluation of non-cooperators is sensitive to their motivation. That is, our participants did not use third-order behavioral information in interpersonal evaluation. Future studies should directly test whether or not the third-order information affects the evaluation of punishment and/or cooperation.

Furthermore, we found that such evaluations were unaffected by whether the punisher had participated in the first stage game (i.e., public goods game). These results are consistent with a previous finding (Mifune et al., 2020), indicating that punishment may be considered undesirable to maintain cooperation. Laboratory experiments have shown that individuals will enforce punishment when it is the only available form of sanction (Yamagishi, 1986; Fehr and Gächter, 2002), yet anthropological studies have found that punishment is not preferred in natural settings (Wiessner, 2005; Guala, 2012). Of the various sanctions that may be used to maintain group order, costly material punishments may not be viewed as the best means (Feldman Hall et al., 2014; Van Doorn et al., 2018; Dhaliwal et al., 2021), especially when the group order is possible to be maintained by implementing exclusion or engaging in targeted gossip (Baumard, 2010, 2011; Eriksson et al., 2021). Additional research is needed to clarify whether the PGG-P context triggers a preference for other forms of sanctions for non-cooperation over costly punishments.

The reason why punishment in TPP is evaluated positively while punishment in PGG-P is evaluated negatively was not elucidated by our study. The current and previous studies (Mifune et al., 2022) have shown that differences in the motivation for non-cooperation and the involvement of the punishers in the first-stage game were not able to explain the differences in punishment evaluations. One remaining possibility is the difference in whether the first-stage game is a group situation with several participants or a person-to-person situation. It would be necessary to examine the differences in the evaluation of punishment by comparing the two situations, with the game type held constant.

Some limitations have to be noted. First, it is sensible to investigate other evaluation aspects. For example, Barclay (2006) reported that punishers were considered trustworthy, but not perceived as nice, while Horita (2010) reported that punishers were more likely to be selected as rewarding participants but less likely to be selected as rewarded by the participants. These findings suggest that punishers may be evaluated differently depending on focal evaluation aspects and dimensions. For instance, the evaluator (participants) may believe that fairness can be restored by punishing fear-based non-cooperation, and may thus evaluate the punishment itself as fair. At the same time, they may believe that punishments for greed-based non-cooperation will prevent continued exploitation and that the punisher is therefore demonstrating leadership (Redhead et al., 2021). Thus, the punishment motive may influence how the punishment is evaluated (Tateishi et al., 2021), with other relevant factors including the form (Eriksson et al., 2021) and subject (Eriksson et al., 2016) of the punishment. Future studies should investigate whether non-cooperation motivations are also related to these factors.

The second limitation is that although we confirmed that the proportion of gender and mean of age were not different between conditions in Study 1, we could not check the sex and age homogeneity between conditions in Study 2. However, as previously argued, the potential influence is considered minimal due to the random assignment method.

Third, we used the third-party PGG-P in Study 2 whereas most previous studies used the second-parity PGG-P (e.g., Kiyonari and Barclay, 2008). One may wonder whether our results can be comparable with previous findings using the second-parity PGG-P. We would like to note that our previous study (Mifune et al., 2020) showed that the standpoint of punishers in the PGG-P (i.e., the second-or third-party) did not influence the evaluation of punishment. Thus, we believe that the use of the third-party PGG-P does not undervalue our implications for the previous studies.

Fourth, our studies did not include direct manipulation checks. In both studies, participants rated their inferred motives of non-cooperators as manipulation checks. The motivation of fear was measured based on the following: “The non-cooperator thought that the other three players would not offer money either, so they should try to avoid being foolish.” The motivation of greed was measured based on the following: “The non-cooperator thought that the other three players would offer money, and thus would have tried to outwit them.” In both studies, there was no significant difference between the degrees of fear and greed in the SIM condition, but the degree of fear exceeded that of greed in the FIRST condition, and the degree of greed exceeded that of fear in the LAST condition (regardless of the game types in Study 2). These results suggest that decision order might have altered the non-cooperation motivation (see the detail in Supplementary materials). However, the phrases “try to avoid being foolish” and “tried to outwit them” may conflate other psychological constructs and the items may not purely capture fear and greed.

The fifth limitation is that the game situation was presented to participants as a scenario with no financial incentive. On the one hand, we agree that directly providing incentives to the evaluation of other’s behavior may help the participants to concentrate on the study. On the other hand, it should be noted that the practices of generating evaluations of others’ behavior and keeping and/or spreading such reputations are something we do on a daily base with no explicit incentives. As what is argued in the evolutionary models and simulations that involve reputation system (e.g., Nowak and Sigmund, 1998), the generation and spread of reputation themselves are effective and need no extra incentive.

## Data availability statement

The datasets presented in this study can be found in online repositories. The names of the repository/repositories and accession number(s) can be found below: The data associated with this study are openly available in (OSF; https://osf.io/chm5p).

## Ethics statement

The studies involving human participants were reviewed and approved by the ethical committee at Kochi University of Technology. The patients/participants provided their written informed consent to participate in this study.

## Author contributions

NM designed the research. NM and YL finalized the design of the experiments, collected the data, analyzed the results, and drafted the manuscript. All authors contributed to the article and approved the submitted version.

## Funding

This work was supported by the JSPS KAKENHI Grant Number 21H00934 for NM, and JST CREST Grant Number JPMJCR21D4, Japan for YL.

## Conflict of interest

The authors declare that the research was conducted in the absence of any commercial or financial relationships that could be construed as a potential conflict of interest.

## Publisher’s note

All claims expressed in this article are solely those of the authors and do not necessarily represent those of their affiliated organizations, or those of the publisher, the editors and the reviewers. Any product that may be evaluated in this article, or claim that may be made by its manufacturer, is not guaranteed or endorsed by the publisher.

## Footnotes

^1^https://www.lancers.jp
