# Supplementary material for: Punishment in the public goods game is evaluated negatively irrespective of non-cooperators’ motivation
Source: Front Psychol. 2023 Jun 29;14:1198797. doi: 10.3389/fpsyg.2023.1198797 (PMC10346865; doi:10.3389/fpsyg.2023.1198797)
Supplement: Supplementary file 1 [file Data_Sheet_1.PDF]

# *Supplementary Material*

# The negative evaluation of punishment in the public goods game irrespective of the non-cooperation motivation

**Yang Li[1 †], Nobuhiro Mifune[2 † *]**

[1]School of Informatics, Nagoya University, Nagoya, Japan

[2]School of Economics and Management, Kochi University of Technology, Kochi, Japan

*** Correspondence:** Nobuhiro Mifune: n.mifune@gmail.com

† These authors share first authorship

## 1 Supplementary Data

## 1.1 Exploratory analysis for study 1

### 1.1.1 Post-experimental questionnaire in Study 1

The thought items were as follows: "The non-cooperator thought that the other three players would not offer money either, so they should try to avoid being foolish," "The non-cooperator thought that the other three players would offer money, and thus would have tried to outsmart them." In both cases, responses were given on a 7-point scale ranging from "1: not at all agree" to "7: very much agree." These items were originally set as manipulation checks. But, according to the definition of fear and greed, we think these were not suitable for the manipulation checks. So, we decided to omit reporting the results in the main text.

Figure S1 shows the mean estimated motivation for non-cooperation in each condition. Our 3 (order condition) x 2 (type of motive: fear or greed) ANOVA with the estimate of the non-cooperation motive set as the dependent variable showed that neither of the two main effects were significant (order: $F(2, 237)=1.51$, $p = .224$, *partial* $\eta^2=.013$; motive: $F(1, 237)=0.42$, $p = .517$, *partial* $\eta^2=.002$), but that the interaction effect was significant ($F(2, 237) = 9.9$, $p < .0001$, *partial* $\eta^2=.077$). The simple main effect of motivation type for each order condition revealed no difference in the SIM condition ($p = .72$, d = .083), while fear significantly outperformed greed ($p < .001$, d = .613) in the FIRST condition, and greed significantly outperformed fear ($p = .006$, d = .48) in the LAST condition.

Supplementary Figure 1. Estimated motivations for non-cooperation in Study 1

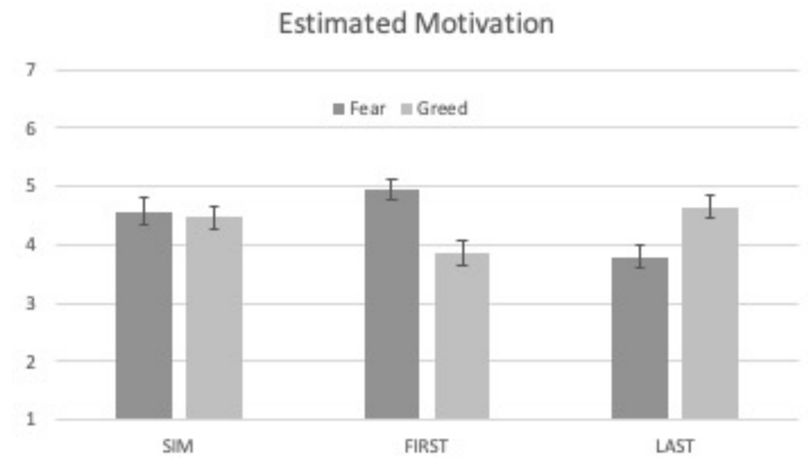


Note: Error bars indicate standard errors.

### 1.1.2 Additional analysis of the main result

Table S1 shows the results of our 3 (order) x 2 (target) x 2 (measurement) ANOVA, in which the difference between impression evaluation and intention to cooperate was set as a within-subject independent variable. Here, we found a significant main effect for target, significant main effect for measurement, and significant interaction between target and measurement. However, there were no significant interactions involving order. Next, our 3 (order) x 2 (target) ANOVA on impression evaluation showed a significant main effect for target ($F(1, 237) = 137.19$, $p < .001$, *partial* $\eta^2 = .367$), non-significant main effect for order ($F(2, 237) = 0.62$, $p = .54$, *partial* $\eta^2 = .005$), and non-significant interaction effect ($F(2, 237) = 0.4$, $p = .670$, *partial* $\eta^2 = .003$). Similarly, our 3 (order) x 2 (target) ANOVA on the intention to cooperate showed a significant main effect for target ($F(1, 237) = 75.48$, $p < .001$, *partial* $\eta^2 = .242$), non-significant main effect for order ($F(2, 237) = 1$ .54, $p = .216$, *partial* $\eta^2 = .013$), and non-significant interaction effect ($F(2, 237) = 0.24$, $p = .79$, *partial* $\eta^2 = .002$). Thus, while target had a stronger effect on impression evaluation than cooperation intention, non-punishers were rated more positively than punishers, regardless of the order in which the non-cooperative behavior was performed during the game.

Supplementary Table 1. ANOVA on impression evaluation and cooperative intention

| IV | df | F-value | p-value | Partial $\eta^2$ |
|---|---|---|---|---|
| Order | 2 | 1.21 | 0.301 | .01 |
| target | 1 | 141.31 | <.0001 | .374 |
| target*Order | 2 | 0.18 | 0.836 | .002 |
| measure | 1 | 273.9 | <.0001 | .536 |
| measure*Order | 2 | 0.98 | 0.378 | .008 |

| | | | | |
|---|---|---|---|---|
| target*measure | 1 | 27.01 | <.0001 | .102 |
| target*measure*Order | 2 | 0.81 | 0.447 | .007 |
| note. df (denominator) = 237 | | | | |

## 1.2 Exploratory analyses for study 2

### 1.2.2 Post-experimental questionnaire in study 2

Figure S2 shows the mean estimated motivations for non-cooperation in each condition. Our 2 (Game) x 3 (Order) x 2 (Type of motive) ANOVA with estimate of the non-cooperation motive set as the dependent variables showed significant main effects for both order ($F(2, 596)=23.76$, $p < .001$, *partial* $\eta^2 = .074$) and game ($F(1, 596)=4.36$, $p = .037$, *partial* $\eta^2 = .007$). There were also significant interaction effects for both type and game ($F(1, 596)=16.35$, $p < .001$, *partial* $\eta^2 = .027$) and type and order ($F(2, 596)=23.98$, $p < .001$, *partial* $\eta^2 = .074$). However, there was no significant main effect for type ($F(1, 596)=1.86$, $p = .173$, *partial* $\eta^2 = .\ 003$), no significant interaction effect for game and order ($F(2, 596)=0.58$, $p = .559$, *partial* $\eta^2 = .\ 002$), and no significant interaction effect for game, order, and type ($F(2, 596)=0.66$, $p = .517$, *partial* $\eta^2 = .\ 002$). The simple main effects of type revealed no difference for the type of motivation in the SIM condition ($p = .834$, d = .021), that fear was higher than greed in the FIRST condition ($p < .001$, d = .59), and that greed was higher than fear in the LAST condition ($p < .001$, d = .376), regardless of game type.

Supplementary Figure 2. Estimated motivations for non-cooperation in Study 2

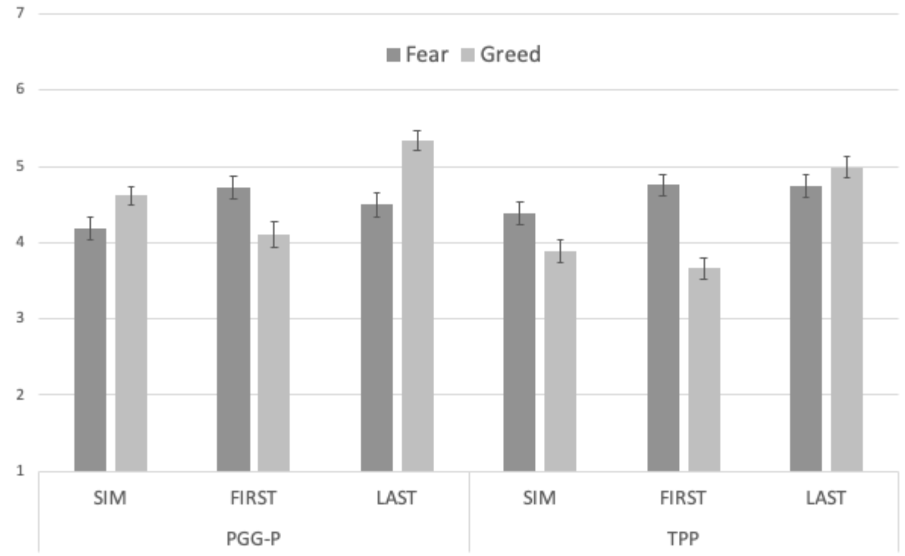


Note: Error bars indicate standard errors.
